# The value of radiographic findings for the progression of pandemic 2009 influenza A/H1N1 virus infection

**DOI:** 10.1186/1471-2334-13-516

**Published:** 2013-11-04

**Authors:** Takanori Funaki, Kensuke Shoji, Nobuyuki Yotani, Tomohiro Katsuta, Osamu Miyazaki, Shunsuke Nosaka, Hidekazu Masaki, Akihiko Saitoh

**Affiliations:** 1 Department of General Pediatrics and Interdisciplinary Medicine, National Center for Child Health and Development, Tokyo, Japan; 2 Department of Medical Subspecialties, Division of Infectious Diseases, National Center for Child Health and Development, Tokyo, Japan; 3 Department of Radiology, National Center for Child Health and Development, Tokyo, Japan; 4 Current address: Department of Pediatrics, St. Marianna University School of Medicine, Kanagawa, Japan; 5 Current address: Department of Pediatrics, Niigata University Graduate School of Medical and Dental Sciences, Niigata, Japan

**Keywords:** Influenza, Chest, Children, Outcome

## Abstract

**Background:**

Most illnesses caused by pandemic influenza A (H1N1) pdm09 virus (A/H1N1) infection are acute and self-limiting among children. However, in some children, disease progression is rapid and may require hospitalization and transfer to a pediatric intensive care unit (PICU). We investigated factors associated with rapid disease progression among children admitted to hospital for A/H1N1 infection, particularly findings on initial chest radiographs.

**Methods:**

In this retrospective study, we investigated the records of children who had received a laboratory or clinical diagnosis of A/H1N1 infection and were admitted to the largest children’s hospital in Japan between May 2009 and March 2010. The medical records were reviewed for age, underlying diseases, vital signs on admission, initial chest radiographic findings, and clinical outcomes. According to chest radiographic findings, patients were classified into 4 groups, as follows: [1] normal (n = 46), [2] hilar and/or peribronchial markings alone (n = 64), [3] consolidation (n = 64), and [4] other findings (n = 29). Factors associated with clinical outcomes were analyzed using logistic regression.

**Results:**

Two hundreds and three patients (median 6.8 years) were enrolled in this study. Fifteen percent (31/203) of patients were admitted to PICU. Among 31 patients, 39% (12/31) of patients required mechanical ventilation (MV). When the initial chest radiographic findings were compared between patients with consolidation (n = 64) and those without consolidation (n = 139), a higher percentage of patients with consolidation were admitted to PICU (29.7% vs.8.6%, P < 0.001) and required MV (17.2% vs. 0.7%, P < 0.001). These findings remain significant when the data were analyzed with the logistic regression (P < 0.001, P < 0.001, respectively).

**Conclusions:**

Consolidation on initial chest radiographs was the most significant factor to predict clinical course of hospitalized children with the 2009 A/H1N1 infection.

## Background

The novel swine-origin influenza A virus first circulated in Mexico in late March 2009 [1] and was confirmed in the United States [2]. It spread globally, leading to an epidemic of influenza in the world, including in Tokyo, Japan, which peaked in October and November, 2009. Risk factors and clinical manifestations associated with pandemic influenza A (H1N1) pdm09 virus (A/H1N1) have been previously reported in adult cases [3-9]. Among children, most illnesses caused by A/H1N1 infection have been acute and self-limited with the highest attack rates, however, some children demonstrated rapid disease progression, required hospitalization, and had to be transferred to pediatric intensive care units (PICUs), and some required mechanical ventilation (MV) [10-14]. It was also demonstrated that this virus caused diffuse alveolar damage in fatal cases [15,16]. Thus, we hypothesized that pulmonary infiltrates on chest radiographs, representing alveolar damage, would be directly correlated with the disease severity. A few studies have described findings on chest radiographs were associated with disease progression of A/H1N1 infection in adults and children [17-19]; however, these studies did not include the information regarding the use of antivirals. In Japan, neuraminidase inhibitors (NIs) have been frequently prescribed in clinical settings [20], and such treatment may affect disease outcome. Thus, we investigated factors associated with rapid disease progression among children admitted for A/H1N1 infections using logistic regression analysis, and focused on initial chest radiographic findings in the context of NI use.

## Methods

### Subjects

This is a retrospective chart review at the National Center for Child Health and Development in Tokyo (the largest children’s hospital in Japan) between May 2009 and March 2010 when A/H1N1 was endemic. The cases were patients who had a history of close contact with family members and/or friends with laboratory-confirmed influenza or those who had received a diagnosis of influenza at our institution. The former cases were diagnosed as A/H1N1 based on their influenza-like symptoms and signs with an appropriate incubation period (within 1–5 days) [20,21]. Patients who were diagnosed by our laboratory had undergone a nasal swab, and a rapid diagnosis using the SPOTCHEM IL SL-4720 (ARKLEY, Inc., Kyoto, Japan). The Espline Influenza A&B-N rapid diagnostic kit (Fujirebio Inc., Tokyo, Japan) was used and was previously found to have a sensitivity and specificity of 96.8% and 97.4%, respectively, for the diagnosis of influenza A [22]. During the study period, 217 inpatients received a diagnosis of A/H1N1 infection, but 14 (6%) were excluded from the study because A/H1N1 infection occurred during hospitalization for a condition other than influenza (n = 7) or because a chest radiograph was not obtained on admission (n = 7), ie, the diagnosis of A/H1N1 infection was confirmed by laboratory testing, but a chest radiograph was not obtained.

### Data collection

Medical records were reviewed for age, gender, underlying diseases, vital signs on admission, oxygen demand, initial chest radiographic findings, time from the onset of illness, dates of visit or hospitalization, discharge dates, and clinical outcomes including PICU transfer and requirement of MV.

### Definitions

Onset of illness was defined as appearance of flu-like symptoms, including fever ≥38°C, cough, headache, chills, myalgia, malaise, anorexia, nausea, vomiting, and diarrhea [23]. Indications for PICU admission were respiratory failure, central nervous system involvement (encephalopathy, status epilepticus, etc.), and abnormal vital signs, namely, ≥2 standard deviations of age-standardized of in heart rate (HR), respiratory rate (RR), and body temperature (BT). Oxygen demand was defined as inability to maintain an oxygen saturation (SpO_2_) ≥93% without supplemental oxygen. Oxygen demand on admission and during hospitalization were defined based on O_2_ requirements at the time of admission and during hospitalization, respectively. Pneumonia was diagnosed based on clinical judgment, which included consideration of respiratory symptoms, physical examination, and chest radiographic findings. Data on serial chest radiographs were collected when available; however, the analyses in the current study were only performed using the initial chest radiographic findings.

### Definition of findings on initial chest radiography

All chest radiographs were read by the pediatric radiology specialists, and their official report was used for analyses. Consolidation was defined as an abnormal increase in radiologic density of an air-filled space, due to accumulation of exudates and tissue debris, as occurs in an infected lung. The extent and distribution of consolidation were also evaluated. Reticulonodularity or diffuse haziness of the lungs was defined as an area of abnormal lung opacity that corresponded to an alveolar, acinar, or amorphous pattern.

### Statistical analysis

All statistical analyses were performed using the SPSS 15.0 software package (SPSS, Inc. Chicago, IL). The chi-square and the Fisher’s exact test were used to compare categorical variables and the Mann-Whitney’s U-test was used to compare numerical variables. Factors associated with the clinical outcomes were analyzed using the logistic regression. Factors that were significant on univariate analysis or were hypothesized to be responsible for clinical outcomes—including age and body temperature—were included in logistic regression analysis. A two-tailed test was used, and P-value of <0.05 was considered to indicate a significant difference.

### Informed consent

Written informed consent for the secondary use of personal medical information was obtained from all patients and/or their parents while patients were hospitalized. This study was approved by the Institutional Review Board of the National Center for Child Health and Development and was performed in compliance with the guidelines set forth by the institutional review board at our institution.

## Results

### Baseline characteristics

In total, 203 patients were enrolled in this study. The baseline characteristics of hospitalized patients are summarized in Table 1. All patients were Japanese, except for two non-Hispanic white patients. Of 203 patients, 139 patients (68%) were diagnosed by the rapid test. The majority of patients (94%) were treated with NIs, but the rest of the patients (6%) were not treated because 1) more than 48 hours had already passed since the onset of illness at the time of admission (4%), 2) they refused to be treated due to fear of adverse effects of NIs (1%), or 3) they were afebrile (1%).

**Table 1:** Baseline characteristics of 203 patients with 2009 A/H1N1 influenza

	**All**	**Group 1**	**Group 2**	**Group 3**	**Group 4**	***P *- values**
	**(N** = **203)**	**(N** = **46)**	**(N** = **64)**	**(N** = **64)**	**(N** = **29)**	
Age (years)						
0–1	15 (7%)	5 (11%)	7 (11%)	2 (3%)	1 (3%)	0.198
1–5	56 (28%)	15 (33%)	20 (31%)	13 (20%)	8 (28%)	
5–10	98 (48%)	15 (33%)	28 (44%)	39 (61%)	16 (55%)	
>10	34 (17%)	11 (23%)	9 (14%)	10 (16%)	4 (14%)	
Gender male	132(65%)	27 (59%)	44 (69%)	45 (70%)	16 (55%)	0.358
Underlying medical conditions						0.004
none	60 (30%)	13 (28%)	16 (25%)	24 (38%)	7 (24%)	
asthma	57 (28%)	8 (17%)	20 (31%)	22 (34%)	7 (24%)	
asthmatic diseases^+^	29 (14%)	2 (5%)	10 (16%)	9 (14%)	8 (28%)	
other respiratory problems^++^	8 (4%)	3 (6%)	2 (3%)	2 (3%)	1 (4%)	
immunocompromised *	4 (2%)	2 (5%)	0 (0%)	2 (3%)	0 (0%)	
others**	45 (22%)	18 (39%)	16 (25%)	5 (8%)	6 (20%)	
Heart Rate on admission	146	138	150	150	150	0.072
(bpm, median) IQR	133-160	100-175	128-172	121-179	128-172	
Respiratory Rate on admission	37	24	40	38	40	< 0.001
(bpm, median) IQR	28-42	14-34	20-60	23-53	17-63	
Body Temperature on admission	38.5	38.7	38.4	38.4	38.2	0.530
(°C, median) IQR	37.9-39.2	37.4-40.0	37.3-39.5	37.0-39.8	36.6-39.9	
SpO_2_ on admission	94	98	96	93	94	< 0.001
(%, median) IQR	93-98	95-100	91-100	88-98	88-100	
O_2_ demand on admission YES	39 (19%)	4 (9%)	9 (14%)	19 (30%)	7 (24%)	0.025
O_2_ demand during admission YES	129 (64%)	10 (22%)	41 (64%)	53 (83%)	25 (86%)	0.002
Time from the onset of illness (hr)						
0–5	38 (19%)	11(23%)	14 (22%)	11 (17%)	2 (7%)	0.976
6–11	27 (13%)	1 (2%)	8 (12%)	13 (20%)	5 (17%)	
12–23	61 (30%)	18 (41%)	14 (22%)	15 (23%)	14 (49%)	
24–47	42 (21%)	11 (23%)	13 (20%)	11 (17%)	7 (24%)	
48 -	20 (10%)	3 (7%)	8 (12%)	9 (14%)	0 (0%)	
Unknown	15 (7%)	2 (4%)	7 (12%)	5 (9%)	1 (3%)	
Systemic steroid use YES	84 (41%)	10 (22%)	24 (38%)	33 (52%)	17 (59%)	0.34
Antibiotics use YES	92 (45%)	5 (11%)	23 (36%)	51 (80%)	13 (45%)	0.10
Antiviral use YES	191 (94%)	42 (91%)	60 (94%)	62 (97%)	27 (93%)	0.994

^+^ Asthmatic diseases were defined as disorders including asthmatic bronchitis and cough variant asthma, but without a diagnosis of asthma.

^++^ “Other respiratory problems” included pulmonary hypoplasia, extralobar pulmonary sequestration, tracheal stenosis, and chronic lung disease.

* “Immunocompromised” was used to indicate patients with nephrotic syndrome, and who were post-living donor liver transplantation for biliary atresia and propionic acidemia.

** Others included chromosomal abnormalities, renal diseases, and congenital heart diseases.

IQR: interquartile range.

The definitions of group 1–4 were as follows:

1) Group 1. Normal finding.

2) Group 2. hilar and/or peribronchial markings alone.

3) Group 3. consolidation; patients with chest radiographs presenting with consolidation, and with or without other findings.

4) Group 4. other findings; patients whose chest radiographs included reticulonodularity or diffuse haziness of the lungs +/− hilar and/or peribronchial markings, hyperinflation, mediastinal emphysema, or subcutaneous emphysema.

The reasons for hospitalization were as follows: respiratory compromise (74.4%, 151/203), neurological issues (14.8%, 30/203), and other reasons (10.8%, 22/203). Respiratory compromise included pneumonia (49.7%, 75/151), respiratory distress (29.1%, 44/151), asthma attack (14.6%, 22/151), plastic bronchitis (inflammatory condition characterized by the presence of bronchial casts typically containing fibrin exudates with varying numbers of inflammatory cells in large airways [2.0%, 3/151]), and other causes (4.6%, 7/151). Neurological issues included febrile seizure (70%, 21/30), febrile delirium (13.3%, 4/30), encephalopathy (6.7%, 2/30), and other issues (10%, 3/30).

**Figure 1:**
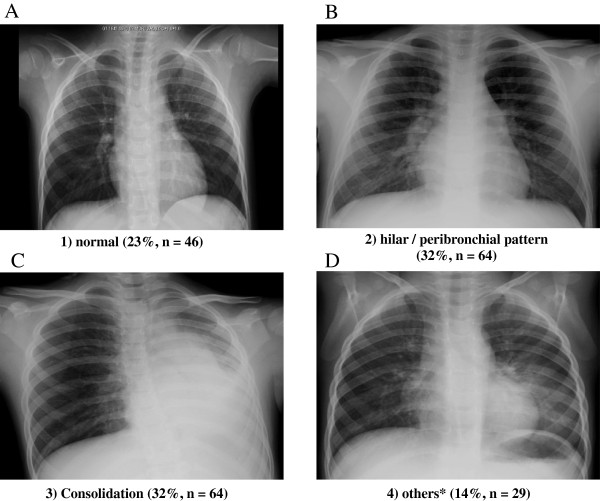
**Representative patterns of the initial chest radiographs. A**. Normal (Group 1), **B**. Hilar and/or peribronchial markings alone (Group 2), **C**. Consolidation pattern (Group 3), **D**. Others (Group 4).

The 203 patients were divided into four groups on the basis of the pattern observed on their initial chest radiographs, which was classified as follows: group 1, normal (22.7%, n = 46); group 2, hilar and/or peribronchial markings alone (31.5%, n = 64); group 3, consolidation (31.5%, n = 64); and group 4, other findings (14.3%, n = 29). Group 2 comprised patients with hilar infiltration and peribronchial markings. Group 3 included patients with chest radiographs showing consolidation, with or without other abnormal radiographic findings. Group 4 included patients with chest radiographs that showed reticulonodularity or diffuse haziness of the lungs, with or without hilar and/or peribronchial markings, hyperinflation, mediastinal emphysema, or subcutaneous emphysema. Representative patterns of chest radiographs for each group are shown in Figure 1.

### Clinical characteristics of patients based on initial chest radiographs

The clinical characteristics of patients based on the initial chest radiographic findings are summarized in Table 1. No significant differences in gender or age were found among the groups. However, the underlying medical conditions were significantly different among the groups; asthma and asthmatic diseases were more common in the patients in group 2, 3 and 4 than in group 1 (P = 0.004). In addition, the respiratory rates in group 2, 3, and 4, were significantly higher than those in group1 (P < 0.001). As expected, the rate of oxygen demand was significantly higher in group 2 (64%, 41/64), group 3 (83%, 53/64), and group 4 (86%, 25/29) compared to group 1 (22%, 10/46) (P < 0.001, P < 0.001, and P < 0.001, respectively). The time from the onset of illness until admission among the four groups was not significantly different (P = 0.98).

### Clinical characteristics of patients based on their clinical course

The clinical characteristics of patients based on their clinical course are summarized in Table 2. No significant differences in gender, age, and underlying medical conditions were found among the groups (P = 0.10, P = 0.12, P = 0.65, respectively). The respiratory and heart rates were also not significantly different among the groups (P = 0.71, P = 0.16, respectively); however, the body temperature was higher in patients admitted to general wards (38.5°C) compared to those admitted to the PICU (38.2°C) or those who required MV (38.0°C) (P = 0.04).

**Table 2:** The patient characteristics based on their clinical course

	**General ward**	**PICU**	**MV**	***P *- values**
	**(N** = **172)**	**(N** = **31)**	**(N** = **12)**	
Age (years)				
0–1	13 (7%)	2 (7%)	1 (8%)	0.10
1–2	8 (5%)	1 (3%)	0 (0%)	
2–5	41 (24%)	6 (19%)	3 (25%)	
5–10	79 (46%)	20 (64%)	8 (67%)	
>10	31 (18%)	2 (7%)	0 (0%)	
Gender male	111 (65%)	21 (68%)	8 (67%)	0.12
Underlying medical conditions				
None	48 (28%)	12 (39%)	3 (25%)	0.65
Asthma	51 (30%)	6 (19%)	5 (42%)	
Asthmatic diseases^+^	27 (16%)	2 (6%)	2 (17%)	
Other respiratory problems^++^	6 (3%)	1 (3%)	1 (8%)	
Immunocompromised*	2 (1%)	2 (7%)	0 (0%)	
Others**	38 (22%)	8 (26%)	1 (8%)	
Heart Rate on admission (bpm, median)	146	147	152	0.71
IQR	136 - 158	126 - 152	132 - 180	
Respiratory Rate on admission (bpm, median)	37	39	39	0.16
IQR	28 - 42	24 - 44	26 - 45	
Body Temperature on admission (°C, median)	38.5	38.2	38.0	0.04
IQR	38.0 - 39.1	37.9 - 39.3	37.2 - 39.1	
SpO_2_ on admission (%, median)	96	93	96	0.05
IQR	93 - 99	92 - 96	90 - 99	
O_2_ demand on admission YES	16 (9%)	24 (77%)	11 (92%)	< 0.001
O_2_ demand during admission YES	118 (69%)	26 (84%)	12 (100%)	0.13
Time from the onset of illness (hours)				
0–5	51 (30%)	4 (13%)	2 (17%)	0.99
6–11	40 (23%)	5 (16%)	3 (25%)	
12–23	66 (37%)	6 (19%)	3 (25%)	
24–47	10 (6%)	9 (29%)	1 (8%)	
48 -	4 (3%)	5 (16%)	3 (25%)	
Unknown	1 (1%)	2 (6%)	0 (0%)	
Antibiotic use YES	72 (42%)	25 (81%)	12 (100%)	< 0.001
Antiviral use YES	160 (93%)	31 (100%)	12 (100%)	0.77

^+^ Asthmatic diseases were defined as disorders including asthmatic bronchitis and cough variant asthma, but without a diagnosis of asthma.

^++^ “Other respiratory problems” included pulmonary hypoplasia, extralobar pulmonary sequestration, tracheal stenosis, and chronic lung disease.

* “Immunocompromised” was used to indicate patients with nephrotic syndrome, and who were post-living donor liver transplantation for biliary atresia and propionic acidemia.

** Others included chromosomal abnormalities, renal diseases, and congenital heart diseases.

IQR: interquartile range.

### Chest radiographic findings and the clinical course

**Figure 2:**
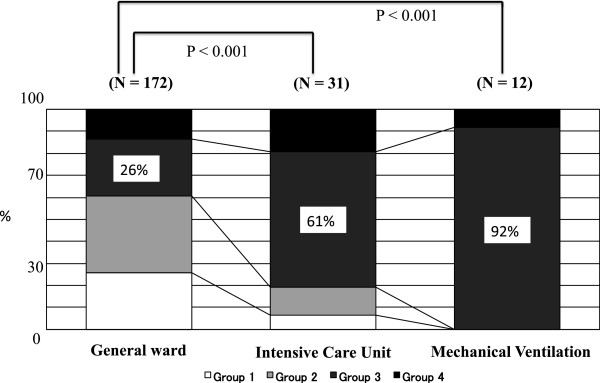
**The patients’ clinical courses after admission based on the chest radiographic findings.** All patients who required mechanical ventilation were admitted to the intensive care unit. Group 1: normal, Group 2: hilar and/or peribronchial markings alone, Group 3: consolidation, Group 4: others.

Of the patients who were admitted to the general ward, 26% (45/172) of patients were categorized in group 3 (consolidation group). In contrast, the percentage of patients with consolidation was significantly higher in patients who were admitted to the PICU (61%) and required MV (92%) compared to those admitted to the general ward (26%) (P < 0.001, P < 0.001, respectively) (Figure 2). When the radiographic findings were divided into those 1) with consolidation (group 3, n = 64) and 2) without consolidation (group 1, 2, and 4, n = 139), a higher percentage of patients (30%, 19/64) with consolidation were admitted to the PICU compared to those without consolidation (9%, 12/139) (P < 0.001). In addition, those with consolidation were frequently required MV (17%, 11/64) compared to those without consolidation (1%, 1/139) (P < 0.001).

In addition, we analyzed the clinical outcomes of patients in group 3 in relation to the extent and distribution of consolidation, as follows: consolidation of ≤25% (20/64, 31%) versus 25-50% (44/64, 69%). Patients with more consolidation (25-50%, n = 42) had worse clinical outcomes: greater proportions required admission to PICU (20/42, 48%) and MV (12/42, 29%) as compared with those with less consolidation (≤25%, n = 22), 1 of 22 of whom were admitted to PICU (5%; P < 0.001) and 0 of 22 of whom required MV (0%; P < 0.001). In addition, patients were classified on the basis of radiographic distribution as unilateral (26/64, 41%) and bilateral (38/64, 59%). The proportions of those admitted to the PICU (8/26 [31%] vs. 13/38 [34%], respectively, P = 0.77) and requiring MV (5/26 [19%] vs.7 /38 [18%], respectively, P = 0.94) did not significantly differ between groups.

Additionally, we performed the analyses in sub-groups of 139 laboratory confirmed patients. Similar to the results of total subjects, the percentage of patients with consolidation (n = 45) was significantly higher in patients who were admitted to the PICU (63%, 12/19) and required MV (86%, 6/7) compared to those admitted to the general ward (28%, 33/120) (P = 0.005, P = 0.005, respectively). When the radiographic findings were divided into those 1) with consolidation (n = 45) and 2) without consolidation (n = 94), a higher percentage of patients (27%, 12/45) with consolidation were admitted to the PICU compared to those without consolidation (7.4%, 7/94) (P = 0.002). In addition, those with consolidation were frequently required MV (13%, 6/45) compared to those without consolidation (1%, 1/94) (P = 0.005).

### Secondary bacterial infections and treatment

Secondary bacterial infection was confirmed by tracheal cultures in only four patients, all of whom required MV. The majority of patients (80%, 51/64) who had consolidation on initial chest radiograph (group 3) received antibiotics without microbiological evaluation. In addition, the percentage of patients (80%) who received antibiotics in group 3 was significantly higher than that in group 1, 2, and 4 (11%, 36%, and 45%, respectively). When the patients were divided by clinical course into those admitted to the PICU (n = 31) and to the general ward (n = 172), a higher percentage of patients who were admitted to the PICU were prescribed antibiotics (84%, 26/31) compared to those who admitted to the general ward (38%, 66/172) (P < 0.001). A systemic corticosteroid was used in 40% (84/203) of patients for asthma or asthmatic diseases, not for the A/H1N1 infection. Overall, the clinical outcome was excellent without any deaths of complications.

### Logistic regression analyses

To investigate the confounding factors impacting on clinical outcomes of the 2009 A/H1N1 influenza infection in patients, the logistic regression analyses were employed, including several important factors contributing to the clinical course, including the patient age, body temperature, consolidation pattern on initial chest radiograph, and oxygen demand on admission. The body temperature and consolidation pattern on initial chest radiographs were independently associated with clinical course of the patients who were administered to the PICU (P = 0.005, P < 0.001, respectively, Table 3A). Additionally, the oxygen demand on admission and the consolidation pattern on initial chest radiograph were independently associated with clinical course of the patients who required MV (P = 0.043, P < 0.001, respectively, Table 3B). Overall, the consolidation pattern on chest radiograph remained the most significant factor to determine the clinical course of patients with the 2009 A/H1N1 influenza infection.

**Table 3:** The results of multivariate analysis for clinical course of the patients

**Covariates**	**Regression coefficient ± SE**	***P *- values**
**A. The patients who were admitted to the pediatric intensive care unit**		
Age	−0.001 ± 0.006	0.889
Body temperature	−0.665 ± 0.239	0.005^+^
Consolidation pattern on chest radiograph	2.002 ± 0.452	< 0.001^+^
O_2_ demand on admission	0.689 ± 0.511	0.177
**B. The patients who required mechanical ventilation**		
Age	−0.016 ± 0.011	0.142
Body temperature	−0.479 ± 0.352	0.174
Consolidation pattern on chest radiograph	3.504 ± 1.093	< 0.001^+^
O_2_ demand on admission	1.468 ± 0.724	0.043^+^

SE: standard error.

^+^ statistically significant.

We further analyzed the data excluding group 1 (patients with normal chest radiograph) to investigate if the consolidation pattern remains a significant factor to determine the clinical course of A/H1N1 infection compared to those with other abnormal chest radiograph. No significant differences except for oxygen demand during hospitalization (P = 0.03) were found among the three groups. When the radiographic findings were divided into those 1) with consolidation (group 3, n = 64) and 2) with other abnormal findings (group 2 and 4, n = 93), a higher percentage of patients (30%, 19/64) with consolidation were admitted to the PICU compared to those with other abnormal findings (13%, 12/93) (P < 0.001). In addition, those with consolidation were frequently required MV (17%, 11/64) compared to those with other abnormal findings (1%, 1/93) (P < 0.001). Similarly, logistic regression analyses demonstrated the body temperature and consolidation pattern on initial chest radiographs were independently associated with the rate of patients who were administered to the PICU (P = 0.017, P = 0.001, respectively). Additionally, the consolidation pattern on initial chest radiograph was independently associated with the rate of patients who required MV (P = 0.001).

## Discussion

Our current study demonstrated that an independent significant factor associated with the deterioration in the clinical course was the consolidation pattern observed on initial chest radiograph in children with the A/H1N1 infection in Japan where NIs have been widely used. The percentage of patients who were admitted to the PICU and required MV was higher among those with consolidation on initial chest radiographs compared to those without. These findings support our hypothesis that the presence of consolidation pattern on chest radiographs representing alveolar damage is directly correlated with the disease severity in children with A/H1N1 infections, even in the area where NIs have been widely used [24-26].

The pattern and extent of consolidation on initial chest radiographs was a significant factor in the clinical course of patients with A/H1N1 infection. Additionally, the body temperature and oxygen demand on admission were independently associated with the clinical course of the patients who were administered in the PICU and who required MV, respectively. This may be related to the fact that the body temperature of patients hospitalized in the PICU might have already been controlled by their having received antipyretics or anesthetics in preparation for MV. Additionally, oxygen demand on admission may reflect the direct involvement of infection in the lung parenchyma, which is closely related to consolidation on chest radiographs. Meanwhile, oxygen demand might have been masked, because certain patients under serious condition had supplied oxygen at the time of SpO_2_ measurement.

Several previous studies of A/H1N1 reported that one of the most important risk factors for complications of severe illness with A/H1N1 infection were age [10,27-29]. In the current study, however, age was not a significant factor associated with rapid disease progression of A/H1N1 infection in children. This discrepancy may be explained by the fact that age distribution of A/H1N1 infection in children in Japan was toward school-aged children compared to infants and younger children [30]. Similar trends were observed in other Asian countries [31], suggesting that the influence of host genetic factors or the existence of maternal cross-reacting antibodies protected infants and younger children. Additionally, the use of NIs may contribute to the difference. In Japan, nearly all patients with laboratory confirmed influenza by the rapid antigen test are prescribed NIs [24]. A further study is warranted to validate these findings.

According to a report, reviewing the chest radiographic findings in patients with A/H1N1 infection demonstrated that the predominant chest radiographic findings in children were interstitial pneumonia (34.3%), followed by consolidation and/or atelectasis (23.8%), normal radiographs (22.3%), lobar or segmental consolidation (19.4%,), and pleural effusion (14.9%) [10]. In addition, several studies reported the findings of chest radiographs and computed tomography (CT), especially in terms of the patterns, distributions, and extent of lung involvement [10,17,32-36]. One study in adults revealed that normal chest radiographs were common among patients presenting to the hospital for A/H1N1 infection-associated symptoms without evidence of respiratory difficulties, but that the factors associated with an increased likelihood for abnormal chest radiographic findings were dyspnea, hypoxemia, and diabetes mellitus [32]. The most common abnormal radiographic finding for lung involvement was a mixture of air-space consolidation and reticulonodularity or diffuse haziness of the lungs with a patchy pattern and lower/middle zone predominance [17,33,35,36].

According to the report from Korea, abnormal chest radiographic findings were uncommon, but the major abnormal findings in chest radiographs and CT images were prominent peribronchial markings and patchy consolidation with mediastinal lymph node enlargement, pleural effusion, and pneumomediastinum in children with A/H1N1 infections [34]. A few previous studies evaluated the relationship between disease progression of A/H1N1 and findings on chest radiographs. Lee *et al.* demonstrated that bilateral, symmetric, and multifocal areas of consolidation associated with reticulonodularity or diffuse haziness of the lungs were the predominant radiographic findings in pediatric patients with a more severe clinical course of A/H1N1 infection [17]; however, limited patient characteristics were reviewed in that study, and no multivariate analysis was performed to evaluate possible confounding factors. A similar trend was described in other two studies, and the findings of a multifocal patchy consolidation pattern with bilateral or diffuse lung involvement on admission should alert of the impending severity of disease [18,19]. The clinical and pathophysiological differences between patients with consolidation and those with reticulonodularity or diffuse haziness of the lungs are likely due to the nature and extent of the inflammation. Consolidation on chest radiographs reflects the presence of alveolar spaces containing varying numbers of neutrophils and mononuclear cells admixed with fibrin and edema fluid, which may lead to airway obstruction, including atelectasis and plastic bronchitis. In addition to primary viral infection, secondary bacterial infection may be associated with consolidation on chest radiographs, because the normal epithelial cell barrier to infection and loss of mucociliary clearance enhance bacterial pathogenesis [23,37-40]. In addition, reticulonodularity or diffuse haziness of the lungs on chest radiographs indicates that inflammation affects the interstitium, and pulmonary opacities appear as airspace, linear, or bandlike opacities, in a nonfocal, patchy, or mottled distribution of varying density. In addition, reticulonodularity or haziness of the lungs on chest radiographs is diffuse and is a radiographic pattern that reflects noncardiogenic pulmonary edema related to sepsis or diffuse pneumonias [40].

In the current study, most of patients received antivirals; those with consolidation on initial chest radiography (97%) and those with other findings (91-94%). Meanwhile, disease severity was significantly higher in those with consolidation than in those with other findings regardless of administration of antivirals. Although clear benefits of early use of NIs in adults have been described in the previous literatures [20], other data in Japan did not demonstrate the differences in timing of NIs treatment between fatal cases and non-fatal, severe cases [41]. However, no data regarding the efficacy of early use of NIs were available in children with A/H1N1. Early administration of NIs might influence clinical course and outcome; however, in the current study, clinical course and outcome had already determined at the time of admission regardless of NIs administration in this population who received NIs.

We acknowledge that there were several limitations to our study. First, this study was a retrospective study, and we only included patients who required admission and whose initial chest radiographs were available. Some patients in the study may have undergone initial chest radiography at outside hospitals, but their chest radiographs were not available. Second, early medical check-ups were available in Japan, and the majority of patients in this study were prescribed NIs during the early stage of the illness. These clinical interventions could have modified radiological findings and clinical course of the disease compared to the data from other countries, where not all patients may have been able to visit the hospital as soon after the onset of illness. Third, secondary bacterial infection may affect clinical course and radiological findings; however, most of the present patients with a consolidation pattern on initial chest radiographs received antibiotics without microbiological evaluation. Fourth, A/H1N1 infection was diagnosed with a rapid test or medical history, without subsequent confirmation from tests such as polymerase chain reaction. Finally, it was impossible to investigate the relationship between radiographic findings and histopathologic findings [42], because none of the patients underwent a lung biopsy.

## Conclusion

The presence of consolidation on initial chest radiographs was the important factor predicting clinical course of children admitted with the A/H1N1 infection in Japan where neuraminidase inhibitors have been frequently used.

## Abbreviations

A/H1N1: Pandemic influenza A(H1N1)pdm09 virus; PICU: Pediatric intensive care unit; MV: Mechanical ventilation; NIs: Neuraminidase inhibitors; HR: Heart rate; RR: Respiratory rate; BT: Body temperature; SpO2: Oxygen saturation; O2: Oxygen; PCR: Polymerase chain reaction; ECMO: Extracorporeal membrane oxygenation; CT: Computed tomography.

## Competing interests

The authors have indicated they have no financial relationships relevant to this article to disclose. All the authors have no conflicts of interest.

## Authors’ contributions

TF, KS, NY and TK contributed to the conception and design of the study. OM, SN, and HM contributed the reading of the chest radiographs, TF, NY, and AS analyzed and interpreted the data. TF and AS drafted the article and all authors revised and approved the final version of the manuscript.

## Pre-publication history

The pre-publication history for this paper can be accessed here:

http://www.biomedcentral.com/1471-2334/13/516/prepub
